# The remains of the D

**DOI:** 10.1002/jgf2.563

**Published:** 2022-05-30

**Authors:** Junki Mizumoto, Taro Shimizu

**Affiliations:** ^1^ Department of Medical Education Studies, International Research Center for Medical Education, Graduate School of Medicine The University of Tokyo Tokyo Japan; ^2^ Department of Diagnostic and Generalist Medicine Dokkyo Medical University Hospital Tochigi Japan

**Keywords:** emergency medicine, hospital general medicine, medical safety, quality in medical care

1

(Case presenter) A 90‐year‐old woman visited our hospital because of appetite loss for the last 2 weeks. The patient lived in a nursing care house, had good communication with caregivers, with retained basic daily activities. She had a good appetite until 2 weeks before, when she became unable to consume only half of her breakfast and most of her lunch and dinner. Besides, she was able to consume as less as 700 ml of water per day. She also complained of fatigue and headache. The nursing care staff admitted that she had bowel movements approximately every 3 days. One week before, the patient visited another hospital and underwent a brain magnetic resonance imaging scan, which only revealed evidence of a previous ischemic stroke. The patient was dozing all day for the last few days, and finally, she was brought to our hospital for further evaluation.

(Discussant) Appetite loss in older people is a frequent problem in primary care, and differential diagnoses must be considered across the bio‐psycho‐social spectrum (see Table 1). Any disorder in the pathway of food can cause anorexia: oral disorders including caries, stomatitis, denture incompatibility, and lack of hygiene care; difficulty in swallowing including neurological disorder and sarcopenia; esophageal and gastric disorders including cancer, ulcer, and motility disorder; and intestinal disorder including obstruction such as constipation. Feeding may be disturbed by various conditions including taste or smell disorder, a decreased amount of saliva, upper limb dysfunction, inappropriate seated position, and visual disturbance. Progressive cognitive decline, depression, paranoia, and inappropriate feeding assistance may also prevent sufficient feeding.

**TABLE 1:** Differential diagnosis of appetite loss in older patients

Gastrointestinal disorders
Environmental conditions affecting appetite
Systemic diseases
Inflammatory diseases (infectious and noninfectious)
Chronic conditions (malignancy, heart failure, and pulmonary diseases)
Electrolyte disturbance
Endocrinopathy
Neuropsychiatric disorders
Drug
Primary anorexia (e.g., CNS medication, digoxin, levodopa, and theophylline)
Secondary anorexia (e.g., anticholinergics, iron, and metformin)

Abbreviation: CNS, central nerve system.

A wide range of systemic conditions can cause anorexia: infectious diseases including pneumonia, pyelonephritis, cholecystitis, and other systemic infection; noninfective inflammatory diseases; cancers and lymphomas; debilitating conditions including chronic heart failure, chronic pulmonary diseases, neurodegenerative disorders, and rheumatic diseases; electrolyte disturbance including sodium, potassium, magnesium, and calcium abnormalities; and endocrinopathy including hyper‐ or hypoglycemia, thyroid disorders, and adrenal disorders. The persistence of only decreased appetite in the course of 2 weeks reduces the possibility of acute systemic inflammatory diseases, while I would not rule out the possibility of insidious inflammatory diseases such as tuberculosis and malignant neoplasms. Polymyalgia rheumatica and vasculitis would not be highly suspected because of the patient's age. Debilitating conditions should be scrutinized, while there are no specific findings so far. Electrolyte, metabolic, and endocrine disorders would be excluded from laboratory testing: these disorders have a wide variety of clinical manifestations and often have clinical expressions that are not apparent at first glance.

Being seated during feeding may be intolerable for patients in this age group with orthostatic hypotension as a result of synucleinopathies, peripheral neuropathies, and age‐related baroreceptor dysfunction. In older patients who cannot complain of pain, loss of appetite can be a trigger for finding a bone fracture.

All older patients with appetite loss should have their prescription reviewed. Agents affecting the central nervous system, including anticonvulsants, antipsychotics, benzodiazepines, and opiates, sometimes cause anorexia in older patients. Digoxin, levodopa, and theophylline also cause anorexia directly. Depression may cause anorexia, and selective serotonin reuptake inhibitors may also cause nausea, diarrhea, and anorexia. Many other drugs can cause secondary anorexia. For example, anticholinergics may cause dry mouth and delirium, leading to appetite loss.

In older patients, there is not always a one‐to‐one correspondence between symptoms and diagnosis. Many older patients have multiple chronic diseases, and, in some cases, several exogenous factors and events and the accumulation of functional decline lead to a complex course of the medical history. It means that too much focus on the main complaint may prevent physicians from finding the real problem in older patients.

(Case presenter) The past medical history included multiple old cerebral infarcts, vascular dementia, primary biliary cholangitis, hypertension, osteoporosis, and insomnia. The patient regularly visited three clinics: neurosurgery, psychiatry, and internal medicine which were commissioned to the nursing home. The patient had a routine blood test once a year. The last test performed 5 months before revealed the following results: the white blood cell count of 3720 cells/μL, the red blood cell count of 228 × 10^4^/μl, the hemoglobin level of 8.2 g/dl, the hematocrit of 24.8%, the platelet count of 10.4 × 10^4^/μl, serum creatinine level of 1.27 mg/dl, and blood urea nitrogen level of 25.5 mg/dl. The patient regularly took the following drugs: clopidogrel 50 mg/d and lansoprazole 15 mg/d prescribed by the neurosurgeon; olanzapine 5 mg/d, ramelteon 8 mg/d, suvorexant 15 mg/d, and eldecalcitol 0.75 μg/d by the psychiatrist; and amlodipine 5 mg/d, magnesium oxide 330 mg/d, ursodeoxycholic acid 300 mg/d, sodium ferrous citrate 200 mg/d, and potassium aspartate 900 mg/d by the internist. The prescription did not change in the last several years.

(Discussant) The patient suffered from two common problems in current geriatric medicine: fragmentation of care and polypharmacy. The patient was prescribed various kinds of drugs, but their side effects may not be regularly checked. High doses of iron had been prescribed for a long time, probably for anemia. If the patient's anemia was caused by iron deficiency, it might have already been corrected. Platelets usually increase in iron deficiency anemia, which was also not consistent with this case. The patient's anemia was surely not caused by iron deficiency, but to another condition such as known cirrhosis or other etiologies. Iron preparation may cause gastrointestinal symptoms. Potassium can also cause dysphagia. This patient's potassium level was not checked regularly, and it was probable that this prescription was potentially inappropriate.

Eldecalcitol and magnesium oxide were prescribed to the patient by different doctors. Activated vitamin D3 agents are effective in preventing osteoporotic fracture, but long‐term use may cause hypercalcemia because vitamin D enhances intestinal calcium absorption and bone mobilization. Vitamin D‐induced hypercalcemia is quite common in Japan. Hypercalcemia leads to arteriole vasoconstriction and then decreases renal blood flow and glomerular filtration rate. Hypercalcemia also suppresses parathyroid hormone (PTH) secretion and then increases bicarbonate reabsorption. If hypovolemia occurs because of general fatigue and altered mental status caused by hypercalcemia, the kidney injury and metabolic alkalosis are maintained and worsened. The risk of drug‐induced hypercalcemia is thus high in older age and chronic kidney disease. The combination of vitamin D and magnesium oxide makes the risk of hypercalcemia much higher. Magnesium ions, which are divalent cations as calcium ions, stimulate the calcium‐efficient sensing receptors and suppress PTH secretion. Magnesium oxide also causes mild alkalemia because of the following pathway. Magnesium oxide is converted to magnesium chloride in the stomach by gastric acid (hydrochloric acid). Magnesium chloride is then converted to magnesium bicarbonate and magnesium carbonate in the intestine by pancreatic juice (sodium bicarbonate). Finally, intestinal absorption of bicarbonate leads to alkalemia. Therefore, the calcium level should be examined in this patient.

(Case presenter) The patient was seated in a wheelchair and looked lethargic. The patient opened her eyes by calling out. She did not say few words. The vital signs were as follows: body temperature was 36.7°C, blood pressure 160/68 mm Hg, heart rate 60 beats per minute, respiratory rate 16 per minute, and oxygen saturation 97% with ambient air. The conjunctiva was no pallor. There was dryness of the mouth. The jugular vein was not distended. A II/VI systolic ejection heart murmur was heard over the second right sternal border. The lung sounds were clear. The abdomen was flat, soft, and not tender. There was no hepatosplenomegaly or lymphadenopathy. Edema was not appreciated in the limbs. No limb abnormal movement was seen. Deep tendon reflexes were normal. Joints, skin, and neurologic examination were normal. Bruits over the carotid arteries, abdominal aorta, renal arteries, and common iliac arteries were not heard. Tenderness was not seen over the muscles in the limbs.

(Discussant) Physical examination revealed slight altered mental status (AMS). The common causes of AMS in older patients which develops for days and weeks include systemic infectious diseases, electrolyte disorders, endocrinopathy, encephalopathy, chronic subdural hematoma, and adverse drug events.

Hyponatremia, hypernatremia, hypocalcemia, hypercalcemia, hypomagnesemia, and hypermagnesemia can cause AMS. Hypernatremia usually occurs in patients who are unable to drink water on their own. The patient took only about 700 ml of water for the last several days and was at high risk of free water depletion. It was possible that anorexia occurred for some reason and was further aggravated by hypovolemia and dehydration. Long‐term use of magnesium oxide may cause hypermagnesemia. If the serum magnesium level exceeds about 5.0 mg/dl, various symptoms may occur: AMS, decreased or diminished deep tendon reflex, bradycardia, nausea, and skin flushing. If the magnesium level is greater than 12 mg/dL, paralysis of respiratory muscle or lethal arrhythmia may happen.

Considering physical examination, there was no evidence of cardiopulmonary or neurological pathologies. At this point, probable differential diagnoses include inflammatory disease with chronic course and no organ‐specific symptoms (tuberculosis and malignant neoplasm), electrolyte disorders, endocrinopathy (thyroid disorders and adrenal disorders), and adverse drug events. The combination of AMS, dryness of the mouth, and appetite loss is consistent with hypercalcemia, and hypercalcemia should also be on the differential list. Severe hyperglycemia must be ruled out because it may develop similar symptoms.

(Case presenter) The laboratory test revealed as follows: hemoglobin level of 7.5 g/dl and the platelet count of 8.2 × 10^4^/μl; albumin level of 2.8 g/dl, measured total calcium level of 12.1 mg/dl, and corrected calcium level of 13.1 mg/dl; magnesium level of 3.2 mg/dl; creatinine level of 2.94 mg/dl; and blood urea nitrogen level of 37.4 mg/dl. Other results are shown in Table 2. Blood gas analysis was not performed.

**TABLE 2:** Laboratory data on admission

Item	Value	Normal range
WBC	3.6 × 10^3^/μl	3.0–8.5 × 10^3^
RBC	208 × 10^4^/μl	378–499 × 10^4^
Hb	7.5 g/dl	10.8–14.9
Ht	22.4%	35.6–45.4
MCV	107.7	85.0–101.0
PLT	8.2 × 10^4^/μl	15.0–36.1 × 10^4^
AST	24 IU/L	8–38
ALT	10 IU/L	4–44
LDH	175 IU/L	106–211
ALP	51 IU/L	104–338
ɤ‐GTP	23 IU/L	4.7–52
T‐Bil	0.3 mg/dl	0.2–1.2
Cre	2.94 mg/dl	0.4–0.8
BUN	37.4 mg/dl	7.0–18.0
Alb	2.8 g/dl	3.4–5.0
CPK	23 IU/L	29–192
Na	145 mEq/L	136–145
K	3.4 mEq/L	3.5–5.1
Cl	102 mEq/L	98–107
Ca	12.1 mg/dl	8.5–10.1
Mg	3.2 mg/dl	1.7–2.6
Fe	90 μg/dl	50–170
Phosphorus	4.2 mg/dl	2.5–4.5
Ferritin	1776 ng/dl	5–157
Vitamin B12	323 pg/ml	233–914
Folic acid	1.9 ng/dl	3.6–12.9
Glucose	109 mg/dl	74–106
CRP	0.06 mg/dl	0–0.30
TSH	1.94 μIU/ml	0.5–5.0
fT4	1.17 ng/dl	0.9–1.7

Abbreviations: WBC, white blood cell; RBC, red blood cell; Hb, hemoglobin; Ht, hematocrit; MCV, mean corpuscular volume; PLT, platelet; AST, aspartate aminotransferase; ALT, alanine aminotransferase; LDH, lactate dehydrogenase; ALP, alkaline phosphatase; ɤ‐GTP, ɤ‐glutamyl transpeptidase; T‐Bil, total bilirubin; Cre, creatinine; BUN, blood urea nitrogen; Alb, albumin; CPK, creatine phosphokinase; CRP, C‐reactive protein; TSH, thyroid stimulating hormone; fT4, free thyroxine.

(Discussant) The patient's calcium level was high enough to explain the symptoms. The common causes of hypercalcemia are hyperparathyroidism and malignancy (e.g., squamous cell lung cancer, breast cancer, adult T‐cell leukemia/lymphoma, multiple myeloma, and metastatic bone neoplasm) and adverse drug events. The patient's macrocytic anemia and acute kidney injury may suggest multiple myeloma. A bone marrow biopsy should be indicated.

Considering multiple etiologies for various findings in older patients than searching for a single etiology may help physicians make an appropriate diagnosis (Hickam's dictum). The patient's activities were limited by the sequela of old cerebral infarction and vascular dementia. Polypharmacy might have decreased her appetite. Vitamin D and magnesium oxide predisposed elevated serum calcium, which was facilitated by her decreased kidney function. All these chronic conditions might have contributed to her appetite loss. A decrease in food intake exacerbated volume depletion, kidney injury, and hypercalcemia. Hypercalcemia then caused altered mental status and volume depletion and kidney injury. In that way, acute exacerbation of chronic hypercalcemic conditions might occur. To verify this hypothesis, correcting dehydration and stopping eldecalcitol and magnesium oxide are recommended at first. If hypercalcemia is corrected and then does not recur after the medication is optimized, the diagnosis of drug‐induced hypercalcemia will be confirmed.

(Case presenter) The patient was admitted, and 2500 ml of isotonic saline per day was administered. Eldecalcitol and magnesium oxide were stopped. On the second day, 2000 ml of isotonic saline was administered, and the patient became fully conscious. On the third day, the patient started to take all dishes. Her activities in daily living, which were deteriorated to the level of not being able to walk, eat, or even say a few words at admission, improved and she was able to take the dishes, communicate well, and maintain her hygiene as before. The measured total calcium level was decreased to 9.5 mg/dl (corrected calcium level of 10.5 mg/dl) and the creatinine level to 1.5 mg/dl. Her PTH level turned out to be 3 pg/ml (reference range: 10–65 pg/ml). PTHrP was not measured. The patient and her family refused further diagnostic tests including bone marrow aspiration, and her pancytopenia remained unchanged. The ward physician informed every clinic in charge of the patient of the patient's health condition and medication, and asked to omit the prescription of eldecalcitol, magnesium oxide 330 mg, sodium ferrous citrate, and potassium aspartate. She was discharged on the day 20, and hypercalcemia did not recur since then.

## 2 DISCUSSION

The patient developed hypercalcemia as a result of inappropriate medication. The patient was old aged and had chronic kidney disease and should have undertaken regular checkups of serum calcium level during the medication of an activated vitamin D3 agent. As mentioned above, the combination of vitamin D and magnesium oxide increases the risk of hypercalcemia. Vitamin D and thiazide are another combination of leading hypercalcemia because thiazide worsens hypovolemia and metabolic alkalosis. The triad of hypercalcemia, acute kidney injury, and metabolic alkalosis, which is caused by vitamin D with magnesium oxide or thiazide, is the same as traditional “milk‐alkali syndrome.”^1^, ^2^ Traditional milk–alkali syndrome refers to hypercalcemia because of an intake of milk and alkalis (e.g., magnesium oxide, sodium bicarbonate, bismuth carbonate) for treating gastroduodenal ulcer.^3^ This therapy was widely used until antihistamine drugs and proton pump inhibitors were introduced. Today, traditional milk–alkali syndrome disappeared, but has changed its form and *remains* as a new‐type “calcium‐alkali syndrome” because *of the* introduction of vitamin *D. (Remember the title of “The Remains of the D.” Regrettably, the author is not Kazuo Ishiguro.)* This syndrome can be confirmed by fewer tests, and physicians can avoid unnecessary laboratory testing by recognizing the syndrome.^4^

Iatrogenic causes should be considered in older patients with loss of appetite. Various drugs cause altered taste or smell (e.g., allopurinol, antihypertensive drugs, and anticholinergics), dry mouth (e.g., anticholinergics, antihistamines, and diuretics), dysphagia (e.g., bisphosphonates, iron, nonsteroidal anti‐inflammatory drugs, and potassium), nausea (dopamine agonists, metformin, and statins), and anorexia (anticonvulsants, antidepressants, antipsychotics, benzodiazepines, and opiates).^5^ Adverse drug events are common, especially in older patients with polypharmacy. Considering it has not been shown that optimizing medication and improving polypharmacy improve patients' clinical outcomes in randomized clinical trials and other studies,^6^, ^7^ physicians should individualize care coordination in seeing older patients with polypharmacy: gathering the best possible medication history, discussing potential deprescribing with patients, developing a specific follow‐up plan, and reviewing patients comprehensively at every visit may be favorable processes.^8^

In this case, the patient had multiple old cerebral strokes and vascular dementia, which limited her activities of daily living. Ferrous citrate and potassium aspartate, which were judged as inappropriate prescription, might have a latent influence on her appetite. Her chronic kidney disease enhanced hypercalcemia, and the resultant decrease in food intake then exacerbated dehydration, kidney injury, and hypercalcemia. This pattern of symptom development is explained by the synergistic morbidity model. This model is described as follows:

In this model, the patient presented with a history of multiple, generally chronic, diseases, each of which contributed to a common, cumulative morbidity. In combination, these diseases caused functional decline below a threshold tolerable to the individual. When this functional threshold was passed, the patient sought medical evaluation.^9^

It is reported that more than half of older patients do not have a specific disease that corresponds directly to the symptoms and signs presented.^9^ Physicians should be aware that a symptom and a disease do not always have a one‐to‐one correspondence in older adults, and the cumulative effects of patients' bio‐psycho‐social factors should be scrutinized.

The patient regularly saw three doctors. The doctors did their practice according to their own specialties, but each care was not coordinated. No one was in charge of the whole part of her daily living and health maintenance. That is, her medical care was fragmented. Fragmentation was defined as “focusing and acting on the parts without adequately appreciating their relation to the evolving whole.”^10^ Even if each professional does the best for a patient, fragmentation of care sometimes has the unintended consequence of making things worse: each care may become inefficient, ineffective, inequal, antiprofessionalized, and deindividualized.^10^ Instead, integrated care maximizes patients' opportunities for health and healing. Care integration against fragmentation is one of the key roles of primary care professionals in today's health care systems.

In summary, we describe the case of hypercalcemia caused by new‐type calcium‐alkali syndrome. Polypharmacy and adverse drug events are common in older patients, and individualized approaches are needed. Clinical reasoning in older patients often requires considering the cumulative effects of bio‐psycho‐social factors. Primary care professionals are expected to have an important role in integrating patients' health care against fragmentation.

## 4 CONFLICT OF INTEREST

The authors have stated explicitly that there are no conflicts of interest in connection with this article.
